# Inside-out flowers of *Lacandonia brasiliana* (Triuridaceae) provide new insights into fundamental aspects of floral patterning

**DOI:** 10.7717/peerj.1653

**Published:** 2016-02-04

**Authors:** Paula J. Rudall, Marccus Alves, Maria das Graças Sajo

**Affiliations:** 1 Comparative Plant and Fungal Biology, Royal Botanic Gardens, Kew, United Kingdom; 2 Dept. Botânica, Universidade Federal de Pernambuco, Recife, Pernambuco, Brazil; 3 Instituto de Biociências, Universidade Estadual Paulista, Rio Claro, São Paulo, Brazil

**Keywords:** *Lacandonia*, Evolutionary transformation, Inside-out flowers, Mycoheterotrophs, Unisexuality, Triuridaceae, Pandanales

## Abstract

**Background and Aims**. A recently described Brazilian species, *Lacandonia brasiliana*, shares with its longer established putative sister species from Mexico, *L. schismatica*, inverted floral patterning (carpels surrounding stamens) that is almost unique among angiosperms. We present a detailed ontogenetic study of *L. brasiliana* for comparison with other members of the tribe Triurideae (Triuridaceae) to explore the possible evolutionary origins of “inside-out” flowers.

**Methods**. Wild-source populations of *L. brasiliana* were compared morphologically and ontogenetically with related species of Triurideae, using light and scanning electron microscopy.

**Key Results**. Relatively few morphological differences separate flowers of *L. brasiliana* and *L. schismatica*. Both species have tepals with late-developing subapical appendages. In both species, the three central (almost sessile) anthers develop precociously with respect to the carpels; the anthers remain closed, and fertilization is achieved via pollen-tube growth from germinating pollen grains of the same cleistogamous flower. Carpels are initiated on fascicles.

**Conclusions**. The close similarity between the two *Lacandonia* species makes it unlikely that they arose independently from two separate homeotic transformation events; they could either represent sister species or two populations of a single disjunct species. Our study underlines the problematic generic and species boundaries within Triurideae. We present an evolutionary scenario of character evolution in Triuridaceae. The inside-out *Lacandonia* flower could have resulted from a stabilized homeotic transformation; this hypothesis is not in conflict with constrasting theories of the origin of the Triuridaceae flower, which coincided with a shift to unisexuality. The unisexual yet highly plastic flowers that are typical of Triuridaceae could have pre-adapted the origin of the extraordinary *Lacandonia* morphology.

## Introduction

*Lacandonia schismatica*, a species of the mycoheterotrophic monocot family Triuridaceae (for authorities see Table 1), has long been the subject of speculation due to its remarkable “inside-out” flowers, in which the carpels surround the central stamens. This inverted floral patterning is almost unique among angiosperms. The only other examples belong to the genus *Trithuria* (Hydatellaceae), which is a water-lily relative and hence an early-divergent angiosperm that is phylogenetically distant from *Lacandonia* (Rudall et al., 2007; Rudall et al., 2009). Both *Lacandonia* and *Trithuria* are also highly unusual in possessing centrifugal carpel development, suggesting that linked developmental features underlie this bizarre floral morphology (Rudall, 2008). Thus, these two taxa appear to break some of the most important ‘rules’ that govern flower structure (Rudall & Bateman, 2010). The apparent reversal of a powerful constraint on floral patterning in *Lacandonia* has led to speculation that its flower could be derived from a highly condensed inflorescence, and could have evolved by fasciation and/or homeosis (cf. Rudall, 2003; Vergara-Silva et al., 2003; Rudall & Bateman, 2006; Ambrose et al., 2006; Rudall, 2008; Álvarez-Buylla et al., 2010; Garay-Arroyo et al., 2012).

**Figure 1:**
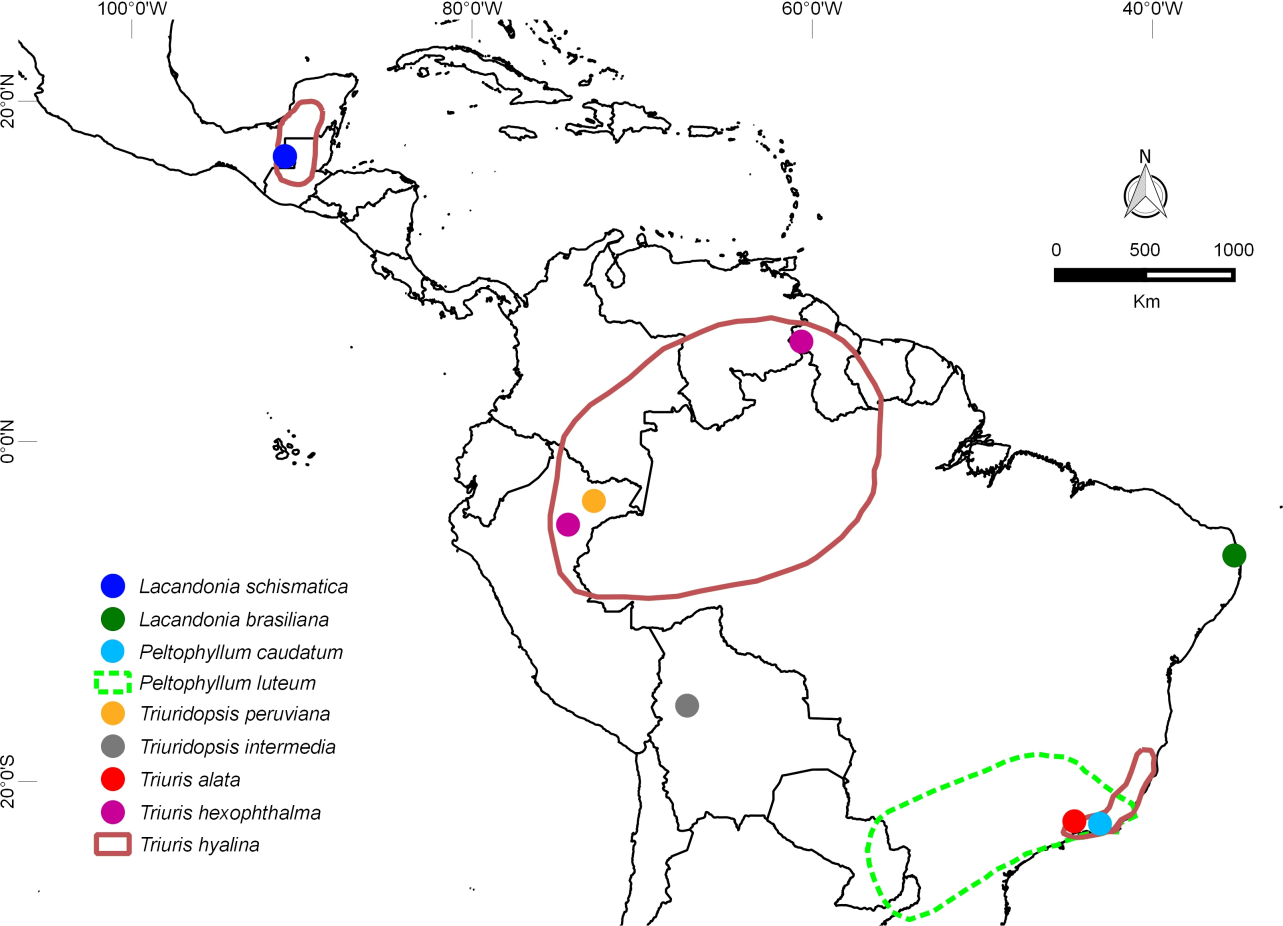
Distribution map. Disjunct locations of species of Triurideae, including the two species of *Lacandonia*, each represented by a few populations. The distribution map was drawn using information available on herbarium specimens and with the species protologue. See text for discussion of taxonomic treatment used (Maas & Rübsamen, 1986; Maas, Maas & Melo, 2015). Note the highly disjunct distributions of *Triuris hexophthalma* and *T. hyalina*. Apart from these taxa and *Peltophyllum luteum*, most other species of Triurideae are narrow endemics.

Until recently, *Lacandonia* was known from a single species (*L. schismatica*) that is endemic to the remote Lacandon rainforest of southeastern Mexico, where it occurs in a few rare and scattered populations (Martínez & Ramos, 1989; Martínez, 1994). The recent discovery of a new species of *Lacandonia* in the extreme north of the Atlantic Forest in northeastern Brazil (*L. brasiliana*: Melo & Alves, 2012), also in rare and scattered populations, raises more questions regarding the evolution of this genus. Specifically, is *L. brasiliana* sister to *L. schismatica*, in which case the genus is more widespread and well-established than was previously thought? Alternatively, did *L. brasiliana* arise independently from a homeotic mutation in a related species from northeastern Brazil? Although a few other Triuridaceae grow in the Brazilian Atlantic forest (six species, three of them Triurideae), most occur in central to southern regions in forest fragments that are relatively humid and shady; *L. brasiliana* is the only species recorded in the northern limit (Fig. 1).

We here present detailed comparative and ontogenetic studies of *Lacandonia brasiliana*. Although these data alone cannot conclusively resolve the long-standing homology questions, they contribute usefully to a monographic background and provide a basis to revisit the ongoing debate. Certainly, a detailed monographic study of Triuridaceae is crucial to understanding character evolution. Taxonomically, Triuridaceae are placed in the order Pandanales, and arranged in three tribes (Table 1): Kupeaeae, Triurideae and Sciaphileae. Morphological and molecular analyses of Pandanales have confirmed monophyly of the tribe Triurideae, to which *Lacandonia* indisputably belongs (Rudall & Bateman, 2006; Mennes et al., 2013).

**Table 1:** Taxa of Triuridaceae.

Tribe	Genera and distribution	Species
Kupeaeae Cheek	Two genera: *Kihansia* Cheek, *Kupea* Cheek & S. A. Williams, Cameroon and Tanzania	
Sciaphileae Miers	Five genera: *Andruris* Schltr., *Hyalisma* Champ., *Seychelleria* Hemsley, *Sciaphila* Blume, *Soridium* Miers, pantropical	
Triurideae Miers	Four genera, South and Central America:	
	*Lacandonia* E. Martínez & Ramos (2 species, Mexico and Brazil)	*L. schismatica* E. Martínez & Ramos *L. brasiliana* A. Melo & M. Alves
	*Triuridopsis* H. Maas & Maas (2 species, Peru and Bolivia)	*T. peruviana* H. Maas & Maas, *T. intermedia* T. Franke, Beenken & C. Hahn
	*Triuris* Miers (3 species, neotropics)	*T. alata* Brade (Brazil), *T. hexophthalma* Maas (Guyana), *T. hyalina* Miers (Mexico, Guatemala, Surinam, Guyana, Venezuela, Colombia, Bolivia (possibly also Peru) and Brazil (disjunct in Brazil; northern states with Amazon forest and southern states with Atlantic Forest). *T. hyalina* encompasses *T. brevistylis* Donn. Sm., *T. major* Poulsen and *T. mycenoides* Ule
	*Peltophyllum* Gardn. (2 species, subtropical and tropical forests of Paraguay, Argentina and Brazil)	*P. caudatum* Poulsen, *P. luteum* Gardner

**Notes.**

Monographic data sources: Cheek (2003), Franke, Beenken & Hahn (2000), Miers (1852), Maas-van de Kamer & Maas (1994), Maas & Rübsamen (1986), Maas-van de Kamer (1995), Maas-van de Kamer & Weustenfeld (1998), Merckx et al. (2013) and Maas, Maas & Melo (2015).

## Materials and Methods

### Material examined

Material was obtained from the following sources: the spirit collection at the Royal Botanic Gardens, Kew (K), the spirit collection at the National Herbarium of the Netherlands, Leiden (NHNL), and wild-source collection (UFP) from Brazil, from a lowland Atlantic Forest fragment with a white sandy soil that is known locally as “tabuleiros.”

Material imaged for this study (for authorities of species names, see Table 1):

*Lacandonia brasiliana*: collected in alcohol in Brazil by one of the authors (MA): UFP: A. Melo et al., 1195, 25 August 2013, Brazil, Paraíba, Reserva Biológica Guaribas, 100–150 msm, 06°42′24″S–35°10′35″W. SisBio collecting permit no. 19769-4 (June 2012–June 2014).

*Lacandonia schismatica*: flowers donated by Dr. Francisco Vergara-Silva, UNAM, Mexico.

*Triuris hexophthalma*: pre-prepared microscope slides made by Traudel Rübsamen-Weustenfeld and housed at the Department of Botany, University of Bochum, Germany). These slides were examined by kind permission of Prof. Thomas Stützel.

*Triuridopsis peruviana* H. Maas & Maas (K: T. Laessoe 265, Peru).

*Peltophyllum luteum* Gardn. (K: Philcox 4245, Brazil).

### Microscopic examination

For light microscopy (LM), flowers were embedded in LR-White resin and sectioned using a Leica rotary microtome. Sections were stained in toluidine blue, dehydrated through an alcohol series to 100% ethanol and then placed in Histoclear. Sections were mounted in DPX mounting medium (distrene, with dibutyl phthalate and xylene). Slides were examined using a Leica DMLB photomicroscope fitted with a Zeiss Axiocam digital camera.

**Figure 2:**
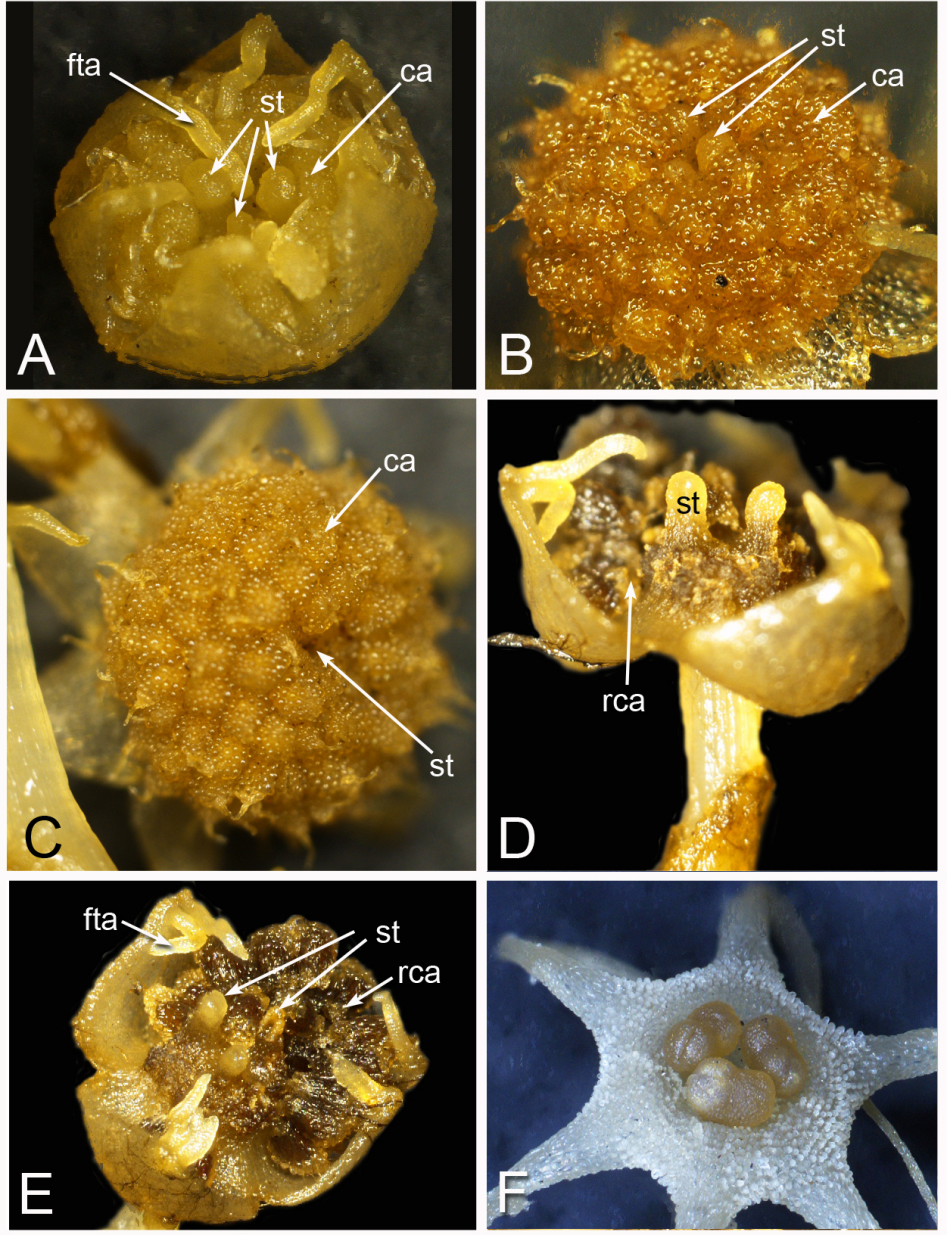
Alcohol-preserved flowers of *Lacandonia brasiliana* (A–E) and *Peltophyllum luteum* (F). (A) Opening flowers showing three central stamens; the filamentous appendages at the tips of the tepals project downwards between the anthers. (B, C) Flowers with open tepals showing three central stamens barely visible, surrounded by carpels. (D, E) Two views of older flower with most fruits dispersed, leaving persistent reduced stamens. (F) Open male flower with three stamens. Labels: ca, carpel; fta, filamentous tepal appendage; rca, remains of carpel (fruit); st, stamen.

For whole mounts, flowers and buds were dissected and dehydrated in an ethanol series. Dehydrated material was critical-point dried using an Autosamdri 815B critical-point drier, dissected where necessary, and mounted onto scanning electron microscope (SEM), stubs using double-sided adhesive tape. Some specimens were imaged using a Nikon Shuttlepix P-MFSC optical system, where necessary subsequently using EDX image stacking to achieve an average focus from multiple primary optical frames (Fig. 2). For scanning electron microscopy, mounted specimens were coated with platinum using an Emtech K550X sputter-coater, and examined under a Hitachi cold-field emission SEM S-4700-II at 2 kV. The resulting images were recorded digitally for subsequent manipulation in Adobe Photoshop. Earlier versions of some SEM images (Figs. 10, 11 and 12D) were published previously by one of us (Rudall, 2003; Rudall, 2008), reproduced here with permission from the *International Journal of Plant Sciences* (University of Chicago Press).

**Figure 3:**
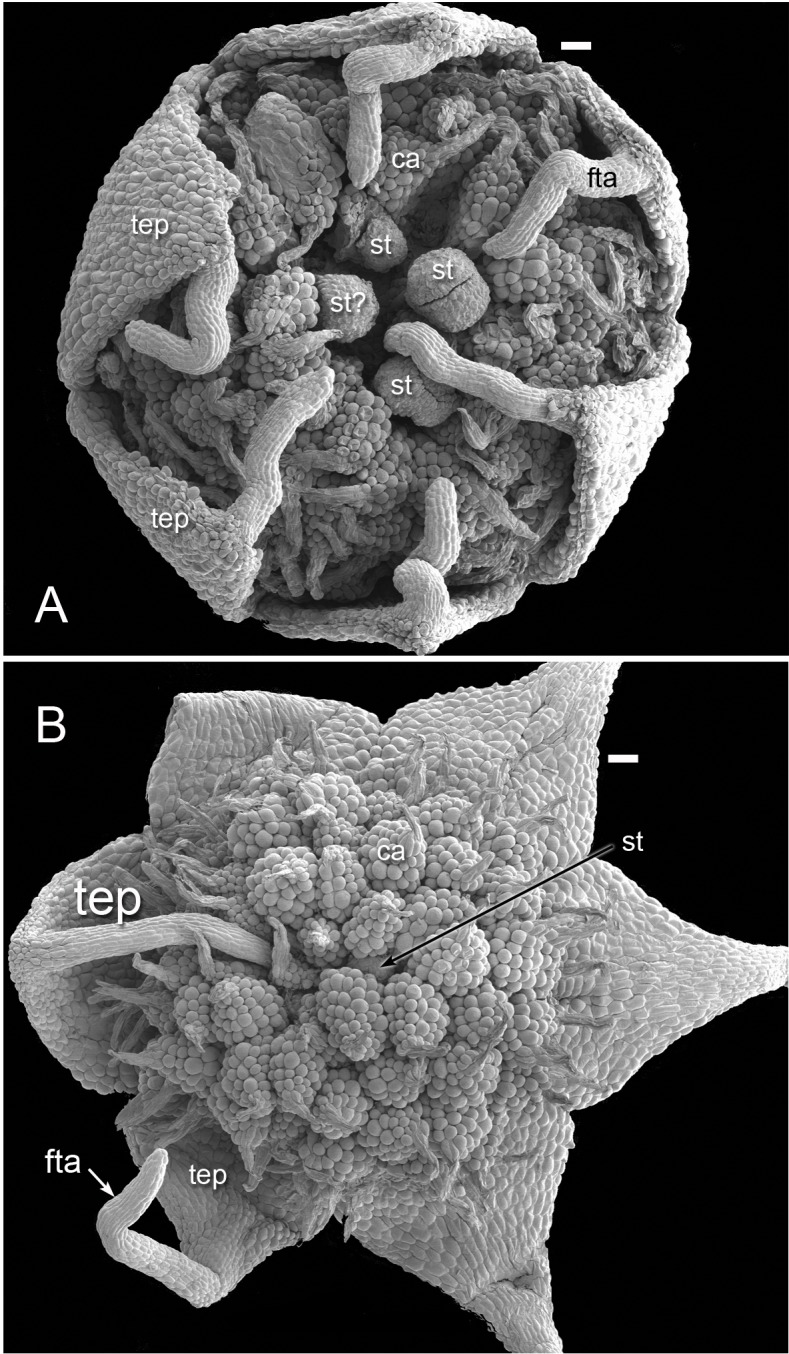
Flowers of *Lacandonia brasiliana* (SEM). (A) Opening flower showing three or four central stamens surrounded by carpels. Tepals with well-developed apical filamentous appendages, their tips inserted between stamens. (B) Open flower with stamen(s) barely visible below surrounding carpels; one of the filamentous tepal appendages still inserted, the rest emerged. Labels: ca, carpel; fta, filamentous tepal appendage; st, stamen; tep, tepal. Scale bars = 100 μm.

**Figure 4:**
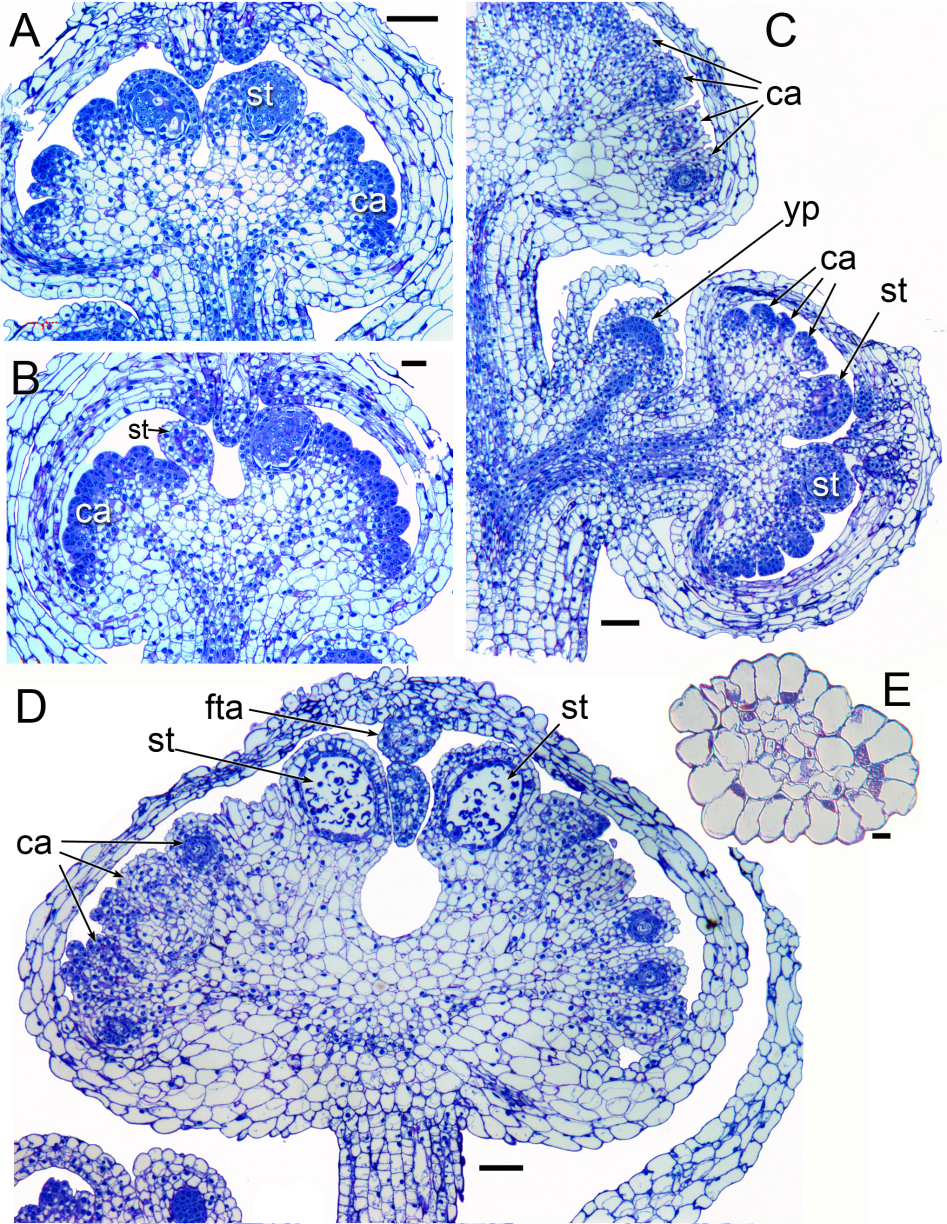
Longitudinal sections of flowers of *Lacandonia brasiliana* (LM). (A, B) Longitudinal sections of unopened buds showing central stamens at a pre-meiotic stage, with anther wall layers already visible surrounding sporogenous cells. The carpels have not yet begun to differentiate ovules. Filamentous appendages have begun to grow from the tips of the tepals. (C) Longitudinal section of inflorescence with three unopened buds at different stages, the youngest flower primordium lacking organs. (D) Longitudinal section of later stage; individual pollen grains visible in anther locules; ovules at megaspore mother stage with developing integuments. Filamentous tepal appendages have extended down between the anthers. (E) Transverse section of an apical tepal appendage showing central vasculature. Labels: ca, carpel; fta, filamentous tepal appendage; st, stamen; yp, young flower primordium. Scale bars: in A−D = 100 μm, in E = 10 μm.

**Figure 5:**
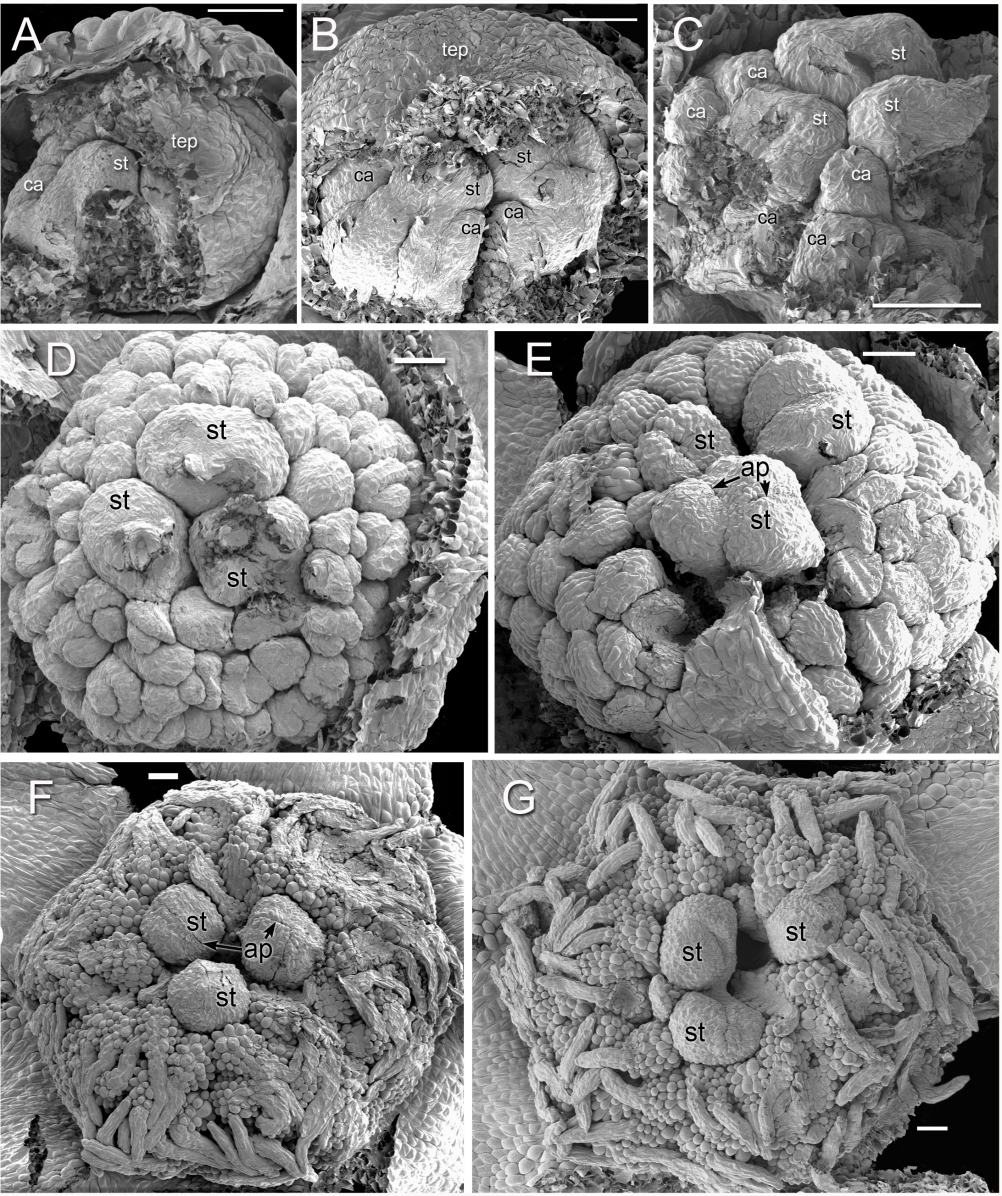
Dissected flower buds of *Lacandonia brasiliana* (SEM). (A–C) Young stages showing developing stamen/carpel fascicles with multiple primordia, of which the central (largest) three will be stamens, and the rest will be carpels. In (A) and (B), the remains of the overlying tepals are still present, before the tepal appendages have grown. (D, E) Mid-developmental stages with three central stamens; carpel tips have not yet extended. In (E) the lowermost anther has three slits and the right-hand ones have two slits. (F, G) Older stages in which the carpel tips have extended. In (F) two anthers are partially fused. Labels: ap, anther slit; ca, carpel; st, stamen. Scale bars = 100 μm.

**Figure 6:**
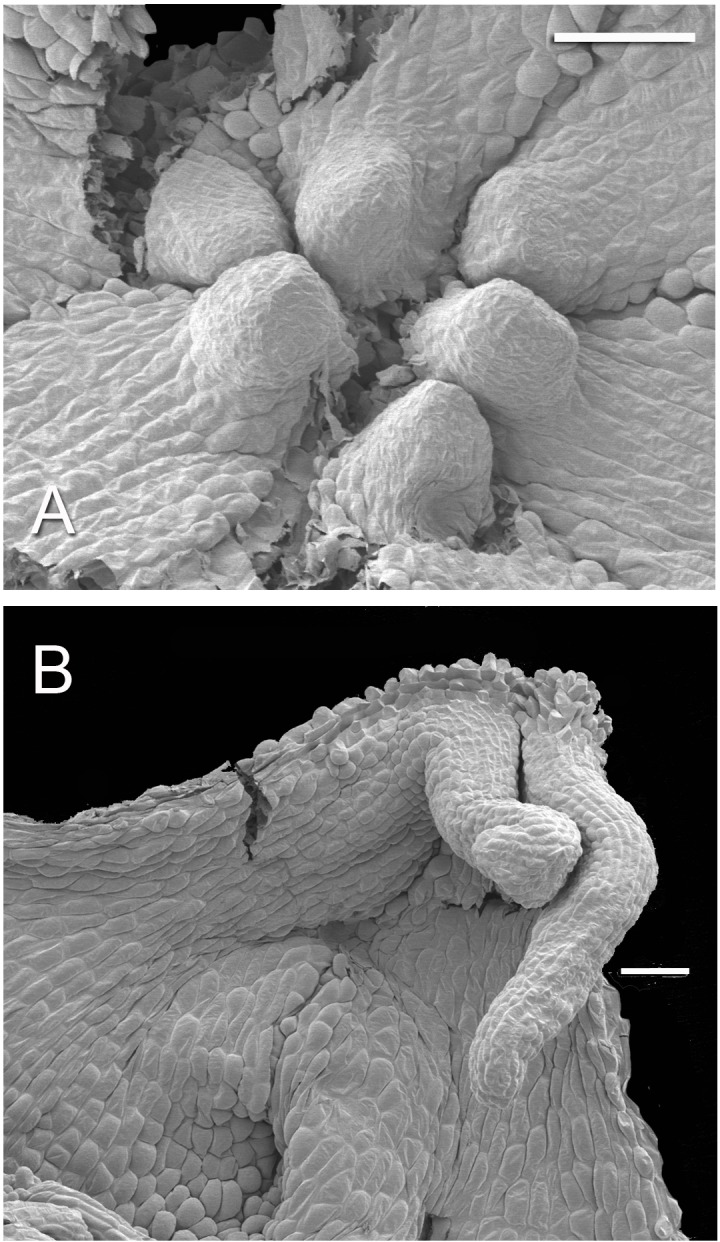
Developing tepal appendages in dissected flower buds of *Lacandonia brasiliana* (SEM). (A) Early development, with tepals fused, viewed from inside. (B) Later stage with filamentous appendages extended between anthers. Scale bars = 100 μm.

## Results

### Flowers of *Lacandonia brasiliana*

Flowers of *L. brasiliana* are bisexual with six tepals arranged in a single whorl. Numerous carpels surround the stamens, which themselves form a single whorl around a central depression (Figs. 2–4). The six tepals are fused at their margins in closed buds (Figs. 5A and 5B) and remain basally connate in open flowers (Fig. 3B). Tepals of open flowers have long filamentous tendril-like structures close to their apices. These subapical filamentous appendages begin to develop in closed buds and grow downwards between the central stamens (Fig. 6). When extended, the tepal appendages consist of a thick epidermal layer with persistent cytoplasmic contents; the epidermis encloses a small amount of thin-walled parenchyma and a central region of vascular tissue (Fig. 4E).

**Figure 7:**
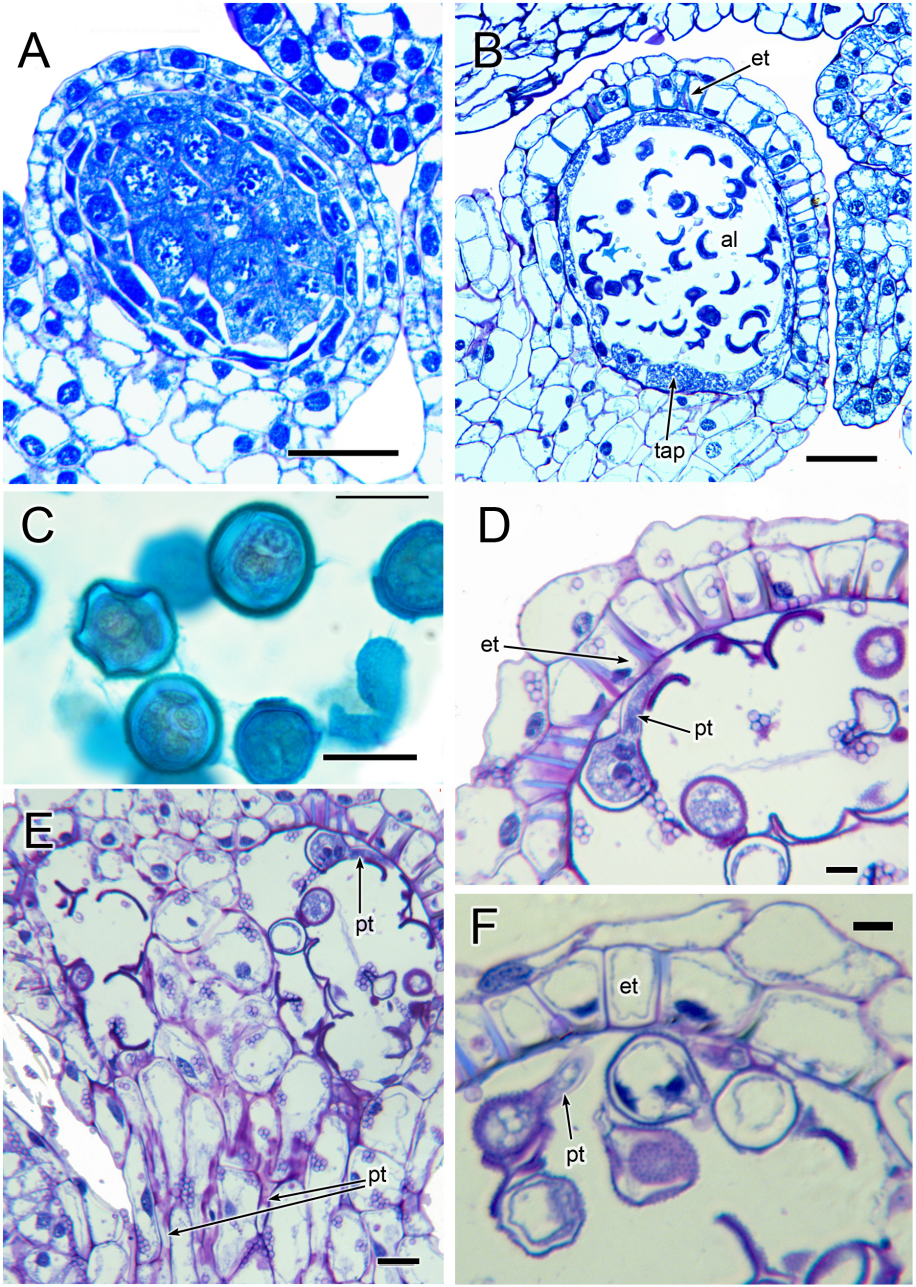
Anthers of *Lacandonia brasiliana* (LM). (A) Longitudinal section of developing anther, with sporogenous tissue in centre and anther wall layers starting to develop. (B) Longitudinal section of mature anther in unopened bud, with individual pollen grains in anther locule and anther wall layers well developed. (C) 2–3-nucleate pollen grains within anther locule. (D, F) Germinating pollen grains inside anther locule. (E) Longitudinal section of mature anther in unopened bud, showing germinating pollen grains in anther locule and pollen tubes in filament. Labels: al, anther locule; et, endothecial layer with wall thickenings; pt, pollen tube; tap, tapetum. Scale bars: in A, B = 50 μm, in D, F = 10 μm, in C, E = 20 μm.

Three (rarely two or four) stamens are present (Figs. 2 and 3), though they are sometimes barely visible below surrounding carpels (Fig. 2B). Development of the stamens is precocious with respect to the carpels. Each stamen consists of an anther borne on a very short filament (i.e., the anther is almost sessile: Fig. 4D). Each anther possesses a slit-like aperture, or sometimes two or three apertures (Figs. 5E and 5F). The anther apertures apparently remain firmly closed, even in open flowers. Occasional anthers appeared partially fused together (Fig. 5G). Early stages possess sporogenous tissue and three to four anther wall layers (Fig. 7A) that subsequently differentiate into an endothecium and tapetum (Fig. 7B). At later stages, the tapetum degenerates and the endothecial cells possess thickenings on their inner walls (Fig. 7D). Mature (three-celled) pollen grains are present in well-developed anthers in unopened buds before the carpels are fully developed (Figs. 4D and 7A–7C). Germinating pollen grains were visible in many anther locules, typically with several pollen tubes growing along the middle lamellae of parenchyma cells in the filament (pollen tubes arrowed in Figs. 7D–7F).

**Figure 8:**
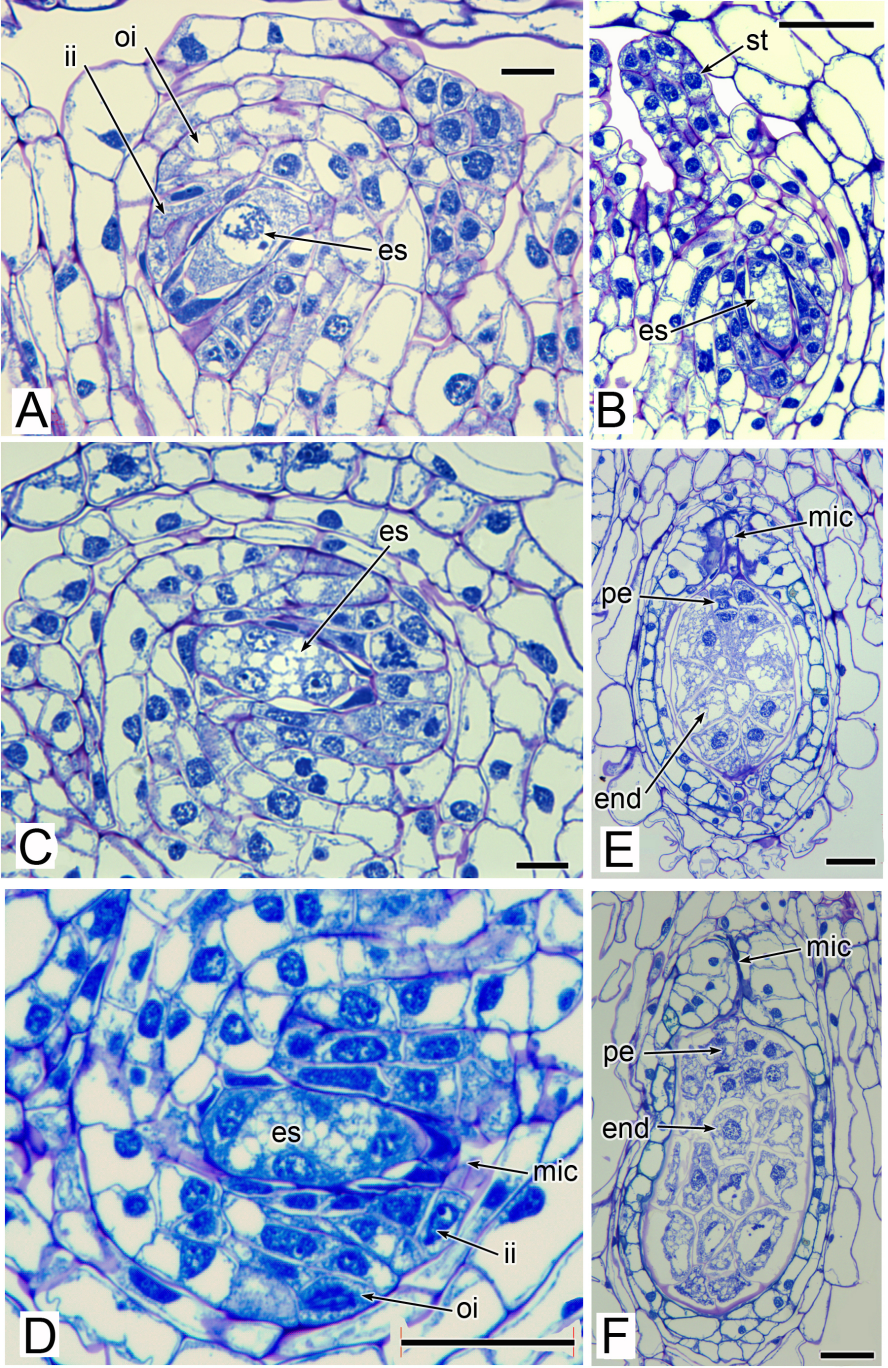
Carpels and ovules of *Lacandonia brasiliana* (LM). (A) Ovule with megagametophyte at megaspore-mother cell stage. (B) Longitudinal section of developing carpel, with megagametophyte at four-celled stage, and filamentous style-like appendage starting to extend. (C, D) Ovules with megagametophyte at four-celled stage. (E, F) Fertilized ovules with proembryo and endosperm. Labels: end, endosperm; es, embryo sac (megagametophyte); ii, inner integument; mic, micropyle; oi, outer integument; pe, proembryo; st, style-like appendage. Scale bars: in A, C = 20 μm, in B, D, E, F = 50 μm.

The stamens are surrounded by numerous carpels with papillate surfaces that make them readily distinguishable from the stamens (Figs. 2 and 3). At early stages the carpels appear as undifferentiated primordia, even as the stamens have begun to differentiate (Fig. 4A). Each carpel possesses a subapical style-like appendage that develops at around the same time as the filamentous tepal appendages (Fig. 5). Each carpel is uniovulate (Fig. 8). The subapical style-like carpel appendages remain unvascularized and lack an obvious transmitting tract (Fig. 8B). The ovules are anatropous and each have two integuments (Figs. 8A and 8D). We observed megagametophytes at the megaspore-mother cell (Fig. 8A) and four-celled stages (Figs. 8C and 8D). In older flowers, many ovules are apparently fertilized and the developing seeds contain endosperm and a proembryo at the micropylar end (Figs. 8E and 8F).

**Figure 9:**
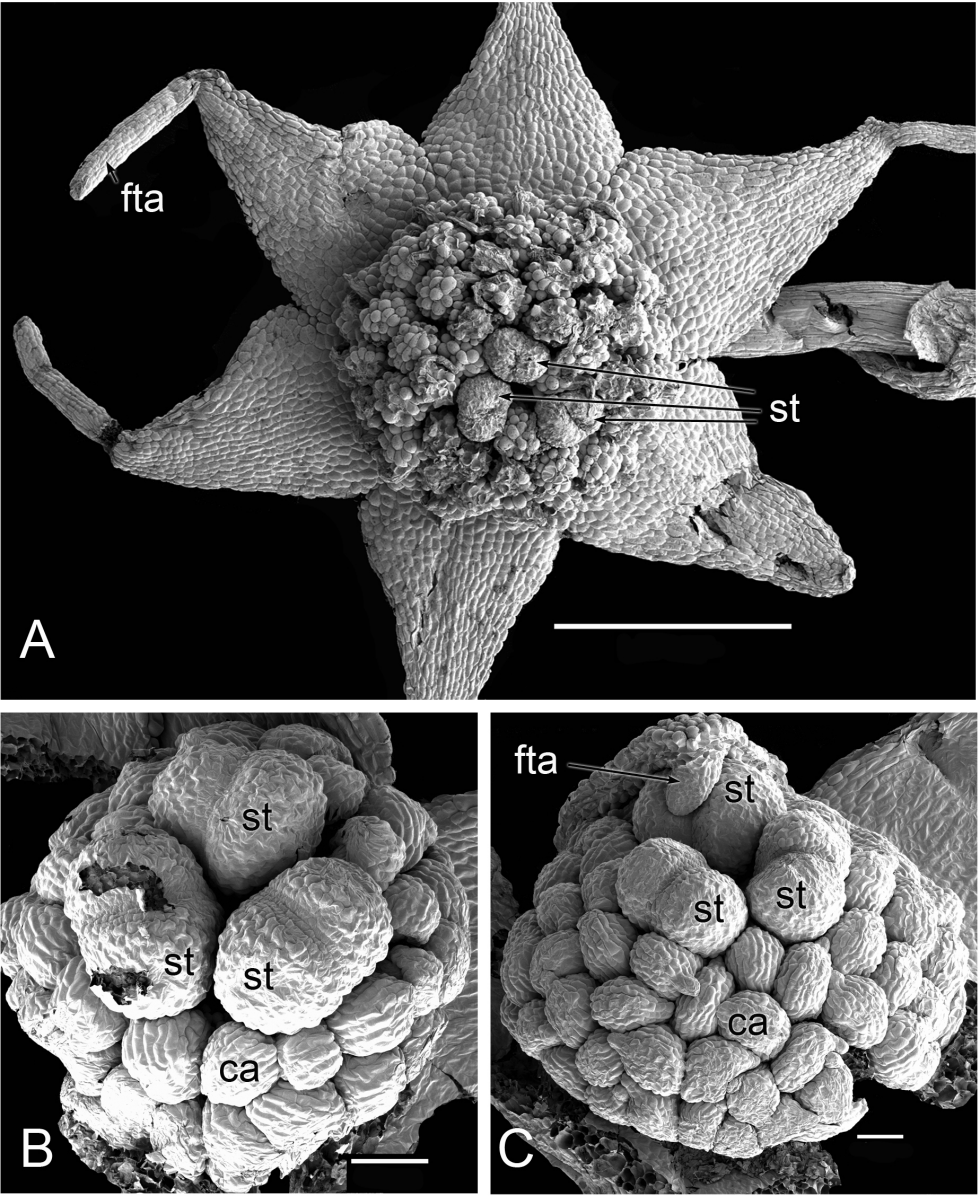
Flowers of *Lacandonia schismatica* (SEM). (A) Open flowers showing three central stamens surrounded by carpels; tepals with well-developed apical filamentous appendages. (B, C) Developing flowers showing three relatively central stamens distal to much smaller developing carpels; filamentous tepal appendage starting to grow in (C). Labels: ca, carpel; fta, filamentous tepal appendage; st, stamen. Scale bars: in A = 1 mm, in B, C = 100 μm.

## Discussion

### *Lacandonia brasiliana* closely resembles *L. schismatica* in flower structure

Our study found remarkably few morphological differences between flowers of *Lacandonia brasiliana* (Figs. 2–8) and *L. schismatica* (Fig. 9), despite the large geographical separation of these apparently isolated species (Fig. 1). Both species possess inside-out flowers characterized by three central stamens that develop precociously with respect to the carpels (Márquez-Guzmán et al., 1989; this paper). Some morphological variation is evident in both species. For example, occasional flowers have reduced or absent stamens (Vergara-Silva et al., 2003; this paper). In both *L. brasiliana* and *L. schismatica*, the tepals have relatively smooth surfaces and are basally connate with late-developing subapical filamentous appendages. Both species have almost sessile anthers that remain closed. Remarkably, in both species fertilization is apparently achieved by pollen tube growth from germinating pollen grains produced in the same cleistogamous flower (Márquez-Guzmán et al., 1993; this paper). The phenomenon of precocious pollen germination within intact anthers, followed by extragynoecial fertilization without pollination, is rare in angiosperms. It has been reported in cleistogamous flowers of some eudicots: a few species of Malpighiaceae (Anderson, 1980) and several species of *Callitriche* (Philbrick, 1984). Among other free-carpellate monocots, pollen-tube growth via the receptacle has also been documented in *Sagittaria potamogetifolia* (Wang, Tao & Lu, 2002). In contrast, pollen grains have not been observed germinating within the same anther locule of other Triuridaceae, such as *Triuris hexophthalma*.

**Figure 10:**
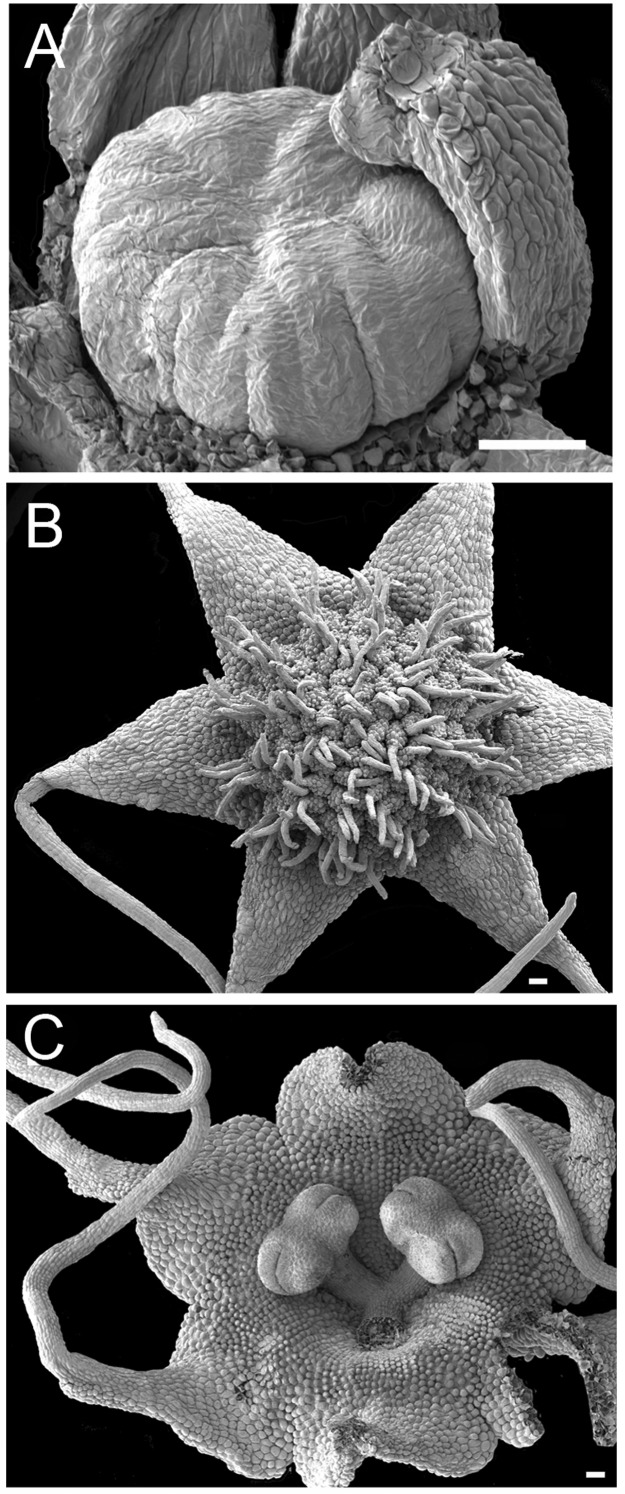
Flowers of *Peltophyllum luteum* (SEM). (A) Dissected young female flower bud with tepals partially removed, revealing central carpellary region before carpel primordia are formed. (B) Open female flower showing carpels in radiating rows with interlocking stigmatic filaments. (C) Open male flower with three stamens (one anther removed). Tepals basally connate with long apical appendages and papillate surface. Scale bars =100 μm. An earlier version of this figure was published previously (Rudall, 2003; Rudall, 2008) and is reproduced here with permission from the International Journal of Plant Sciences (University of Chicago Press).

One possible difference is that the subapical tepal appendages appear shorter in *L. schismatica* than in *L. brasiliana*, but this apparent distinction requires quantification. Tepal appendages are also relatively short in *Triuridopsis peruviana* (Maas-van de Kamer & Maas, 1994). Melo & Alves (2012) observed that *L. brasiliana* differs from *L. schismatica* in having solitary flowers or inflorescences with up to six flowers (compared with up to three in *L. schismatica*), but this feature requires improved population analysis and statistical tests. There is also a potential phenology difference, although this could simply reflect contrasting latitudes: *L. brasiliana* typically flowers in August and September, compared with November and December in *L. schismatica*. These characteristics require more detailed comparative data, both morphological and molecular, to determine whether they adequately delimit *bona fide* species boundaries.

**Figure 11:**
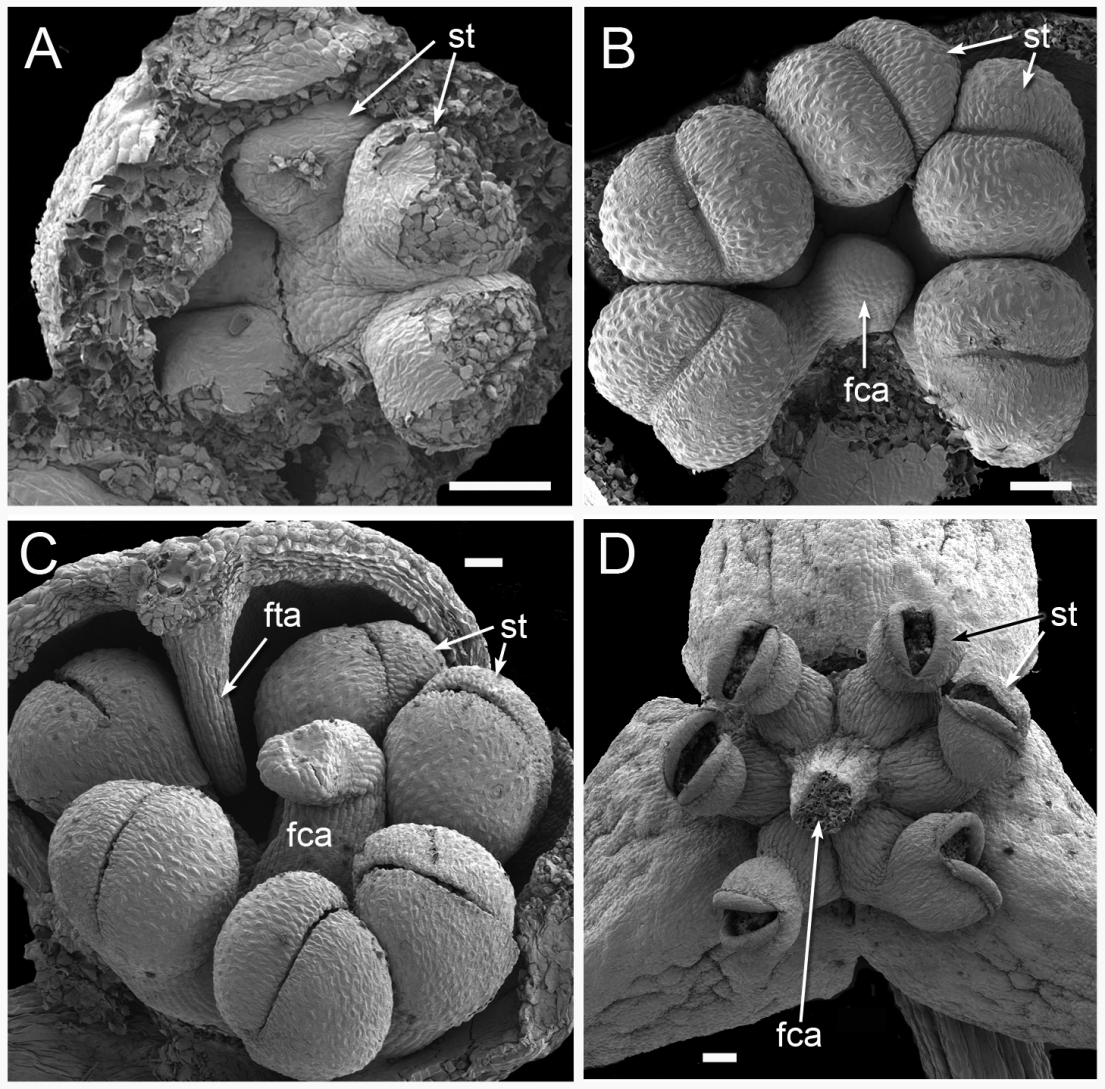
Male flower development in *Triuridopsis peruviana* (SEM; A–C dissected unopened buds, D open flower). (A) Dissected young flower bud with tepals partially removed; filamentous structures absent at this stage; three pairs of developing half-anthers visible. (B) Later stage with filamentous central appendage starting to emerge in flower centre. (C) Filamentous central appendage further extended upwards; also filamentous tepal appendages extending downwards between anthers. (D) Open flower with dehisced anthers and (broken) filamentous central appendage. Labels: fca, filamentous central appendage (broken in D); fta, filamentous tepal appendage; st, stamen (arrows point to two anther halves). Scale bars = 100 μm. An earlier version of this figure was published previously (Rudall, 2003; Rudall, 2008) and is reproduced here with permission from the International Journal of Plant Sciences (University of Chicago Press).

**Figure 12:**
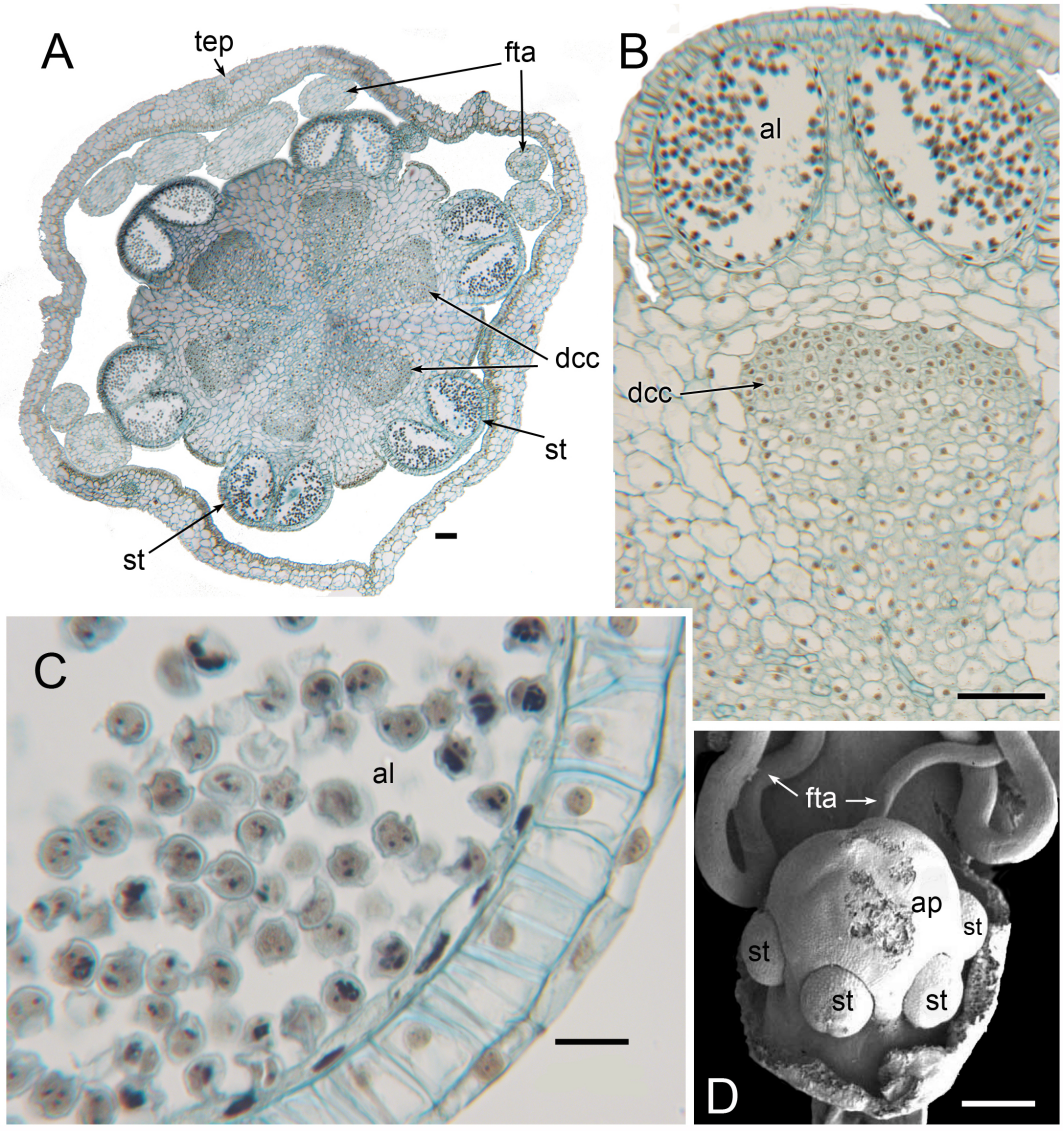
Male flowers of *Triuris hexophthalma* (A–C LM of transverse sections, D SEM). (A) TS unopened flower bud through androphore. (B) Detail of anther and underlying densely cytoplasmic region. (C) anther locule containing pollen grains. (D) Open male flower with prominent central androphore and six sessile half anthers. Labels: al, anther locule containing pollen grains; ap, androphore; dcc, region of cells with dense cytoplasmic contents; fta, filamentous tepal appendage; st, half anther; tep, tepal. Scale bars: in A, B and D = 100 μm, in C = 20 μm. An earlier version Figure 12D was published previously (Rudall, 2003; Rudall, 2008) and is reproduced here with permission from the International Journal of Plant Sciences (University of Chicago Press).

At least some of the features shared between *L. brasiliana* and *L. schismatica* are also common to other species of the neotropical tribe Triurideae (Figs. 10–12). However, the close similarity between the two *Lacandonia* species makes it appear unlikely that they arose independently from two separate homeotic transformation events. Thus, they could either represent sister species that border on the definition of cryptic species, or two populations of a single disjunct species. In either case, the genus *Lacandonia* is more widespread than was previously believed. Such disjunct distributions are not unknown, especially in mycoheterophs, which are inconspicuous and ephemeral and hence are undoubtedly seriously under-recorded (Merckx, Smets & Specht, 2013). For example, the widespread species *Triuris hyalina* is known from disjunct populations in southeast Brazil, the Brazilian Amazonas region and Central America (Fig. 1), encompassing the previously named taxa *T. brevistylis*, *T. major* and *T. mycenoides* (Maas & Rübsamen, 1986; Maas, Maas & Melo, 2015). Interestingly, Vergara-Silva et al. (2003) postulated that *L. schismatica* could have originated from mutations in populations of “*T. brevistylis*” within the Mexican Lacandon rainforest (see also Mabberley, 2008; Espinosa-Matías et al., 2012). “*Triuris brevistylis*” itself represents populations from the Yucatán Peninsula (Guatemala and Mexico) of the widespread species *T. hyalina*. Although “*T. brevistylis*” is typically dioecious or monoecious, Vergara-Silva et al. (2003) observed occasional bisexual flowers with varying positions of stamens and carpels, including homeotic variants possessing inside-out bisexual flowers. It is also noteworthy that most species of Triurideae are narrow endemics known from a single locality or a few localized populations (Fig. 1 and Table 1).

### Taxonomic boundaries within Triuridaceae–Triurideae are ambiguous, though the tribe has two clear morphological synapomorphies

The neotropical tribe Triurideae, which is monophyletic in both molecular and morphological studies (Rudall & Bateman, 2006; Mennes et al., 2013), consist of four genera: *Lacandonia*, *Triuridopsis*, *Triuris* and *Peltophyllum* (Table 1). The tribe is apparently well-defined by two morphological synapomorphies: (1) the long filamentous appendages that grow from the tepal tips at relatively late stages of development, and (2) carpel orientation and development (i.e., carpel fascicles—structures that are discussed in more detail in the next section). Both of these remarkable features occur in all species of Triurideae, including *Lacandonia brasiliana*. The developmental timing and anatomy of the subapical tepal appendages, which occur in both male and female flowers of unisexual-flowered Triurideae, suggest that they are osmophores (Rudall, 2003; Rudall, 2008). Filamentous appendages are common in sapromyiophilous mycoheterophs that produce otherwise inconspicuous flowers (Vogel, 1990). Although filamentous structures are common in general in the family Triuridaceae, in species of tribes Sciaphileae and Kupeaeae they grow from different parts of the flower, such as the bases of the tepals in male flowers of *Seychellaria* (Maas-van de Kamer & Weustenfeld, 1998; Rudall, 2008). The epidermal hairs at the tepal tips of some *Sciaphila* species (e.g., *S. picta*: Maas & Rübsamen, 1986) are non-homologous with the relatively large, vascularized and organ-like appendages of Triurideae.

Our study underlines the problematic generic and species boundaries within Triurideae. Existing molecular data do not resolve this issue. The molecular analysis of Mennes et al. (2013) placed *Lacandonia schismatica* (using the single available plastid sequence, *atpA*) as sister to *Triuris* (*T. hexophthalma* plus *T. hyalina*), with relatively long branch lengths between taxa. Population-level studies are needed to resolve the systematics of *Lacandonia*, including species of *Triuridopsis* and *Peltophyllum* and improved sampling within *Triuris*, perhaps focusing on nuclear gene regions.

In terms of flower structure, *Peltophyllum* (Figs. 2F and 10) is arguably the least complex genus of the tribe Triurideae because its male flowers lacks the central appendage that characterizes male flowers of *Triuridopsis* and *Triuris* (Figs. 11 and 12). The remarkable ovoidal or conical central structure in *Triuris* was termed an androphore by Maas & Rübsamen (1986). The term “androphore” normally indicates a central column formed by united filaments in a male flower. We use this term here for convenience, though there is no evidence that this unusual structure is formed from united filaments in *Triuris*. In some other Triuridaceae the filaments are united at their bases. The androphore is late-developing, at least in “*Triuris brevistylis*” (Vergara-Silva et al., 2003; Espinosa-Matías et al., 2012). It contains enigmatic regions of cells with dense cytoplasmic contents (Fig. 12B) and is most likely a scent-producing structure (osmophore: see also Rübsamen-Weustenfeld, 1991; Rudall, 2003). Such retarded development is typical of structures that are additional to the normal floral organ complement, such as many osmophores and some types of nectary (Rudall, 2010).

The Peruvian species *Triuridopsis peruviana* was segregated from *Triuris* based primarily on two characters. Firstly, its anthers possess filaments, compared with sessile anthers in *Triuris*. Secondly, it has a sterile projection in the centre of the male flower instead of an androphore (Maas-van de Kamer & Maas, 1994); this appendage appears to emerge directly from the receptacle (Figs. 11B and 11D). Subsequently, Franke, Beenken & Hahn (2000) added a second species of *Triuridopsis*, *T. intermedia* from Bolivia, which differs from *T. peruviana* in possessing a relatively short central appendage. However, the androphore itself appears highly plastic. In “*Triuris brevistylis*,” Vergara-Silva et al. (2003) documented male-like flowers with a malformed or arrested androphore and female-like flowers lacking an androphore entirely and possessing intermingled anthers and carpels, or even inside-out flowers. On the other hand, they found no floral variants of *Lacandonia* that possessed an androphore, and we also found none in our material. Thus, existing comparative morphological data are conflicting, but the discovery by Melo & Alves (2012) of *Lacandonia* in northern Brazil opens exciting prospects, even making more plausible the possibility of a close relationship with *Peltophyllum*, which occurs in Paraguay, Argentina, and south-western Brazil (Fig. 1 and Table 1).

### New insights on character evolution in Triuridaceae

**Figure 13:**
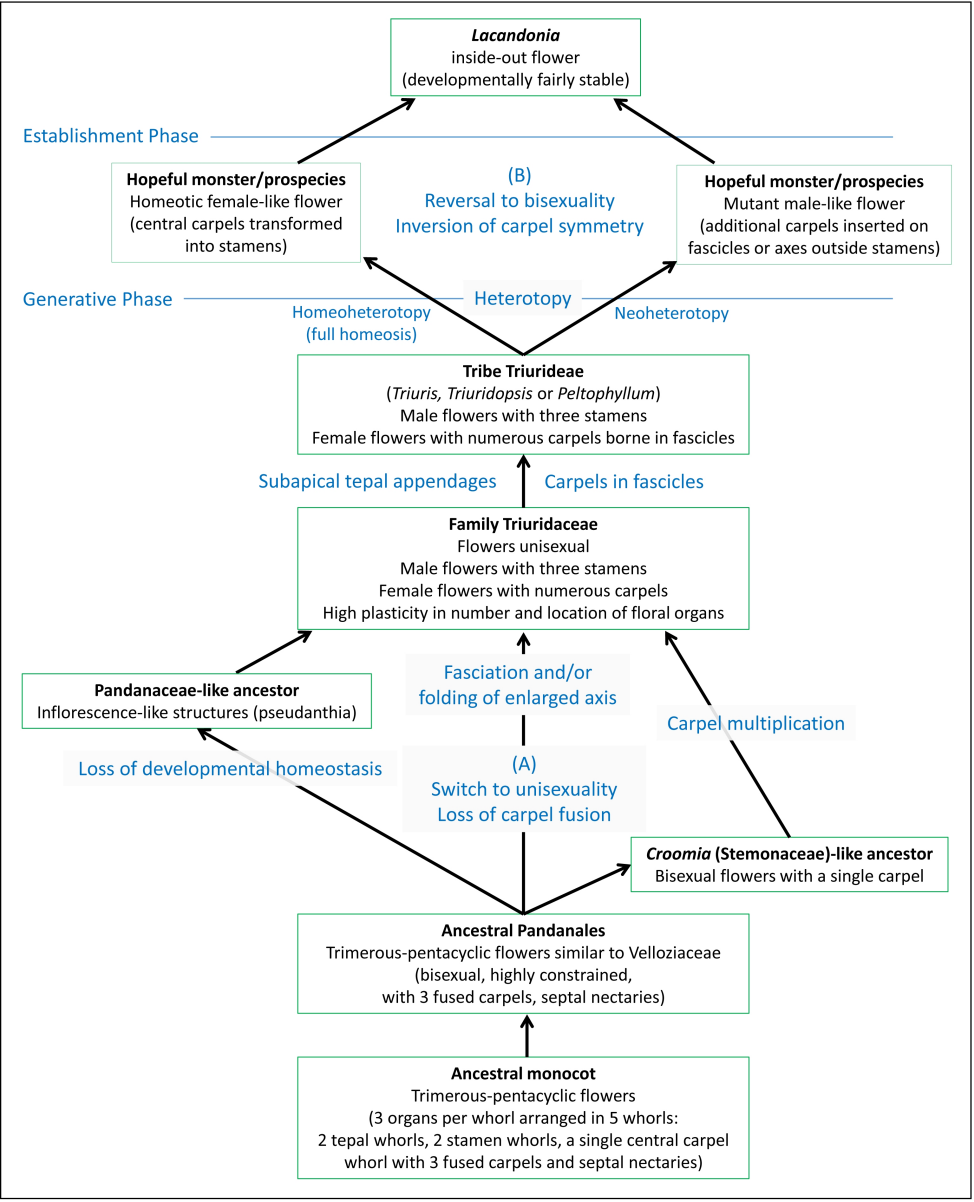
Diagram illustrating separation of different phases leading to the inside-out *Lacandonia* flower. The evolutionary scenario includes contrasting hypotheses at two levels: (A) transformation from ancestral Pandanales to unisexual Triuridaceae, leading to and potentially preadapting (B) transformation from a *Triuris*-like ancestor to bisexual *Lacandonia*. Regarding process terminology, heterotopy represents a spatial shift in a developmental programme and its resulting phenotypic structure, homeoheterotopy is the partial or complete replacement of a pre-existing structure by a contrasting structure, and neoheterotopy is the shift of a structure to a new location not previously occupied by an existing structure (Bateman & DiMichele, 2002; Baum & Donoghue, 2002).

The debate on the evolutionary origin of the inside-out *Lacandonia* flower has become entangled in multiple issues. To clarify the problem, we present an evolutionary scenario (Fig. 13) that illustrates the different phases that led to this bizarre phenotype. Contrasting hypotheses are applicable to at least two levels in this process.

To work backwards from the *Lacandonia* phenotype, the arguments for homeotic transformation of organ types appear highly plausible, especially given that studies of the B-function (Ls-AP3 and Ls-PI) and C-function (Ls-AG) MADS-box floral-organ identity genes show that B-function occurs in the flower centre in *Lacandonia* (Álvarez-Buylla et al., 2010). Homeotic transformations are certainly common within Triuridaceae, and could have caused a saltational origin of *Lacandonia* from a “hopeful monster” (cf. Bateman & DiMichele, 2002). Hemsley (1907) noted in *Sciaphila* (Triuridaceae) that even in species bearing typically unisexual flowers, many are “quasi-hermaphrodite” and often irregular as to the number and location of the floral parts. He commented on Triuridaceae in general that “it would appear that these small flowers are peculiarly subject to disturbances in their development” (page 75). However, as shown in Fig. 13, it remains ambiguous whether the homeotic transformation in *Lacandonia* resulted from a replacement of carpels with stamens in a female-like flower (homeoheterotopy) or *de novo* origin of carpels in a new location in a male-like flower (neoheterotopy: Bateman & DiMichele, 2002; Baum & Donoghue, 2002). Indeed, since both male and female flowers occurred in the putative ancestor, it is difficult to see how developmental-genetic studies that inevitably investigate a snapshot in evolutionary time could conclusively distinguish between these processes.

As discussed in the previous section, two clear synapomorphies place *Lacandonia* firmly within the neotropical tribe Triurideae: filamentous subapical tepal appendages and the highly unusual (possibly unique) carpel fascicles, which have also been subject to contrasting interpretations. In all species of Triurideae, including both species of *Lacandonia*, the carpels are arranged on ridges in radial double rows (Vergara-Silva et al., 2003; Rudall, 2008; this paper). This feature is sometimes more clearly discernible at early developmental stages, as the ridges are more disorganized in older buds (Figs. 5D, 9C and 10B). Organ primordia develop along the ridges, maturing from the inside outwards. In other Triurideae, all of these organ primordia become carpels, but in *Lacandonia*, the apices of the three primary ridges develop into stamens. Rudall (2008) compared the ridges (termed carpel fascicles) with stamen fascicles, which occur widely in angiosperms, including some species of the related family Velloziaceae (Sajo, Mello-Silva & Rudall, 2010). Sokoloff et al. (2007) suggested that the carpel fascicles or ridges result from folding of an enlarged (perhaps fasciated) floral apex (see also Rudall, 2008; Endress, 2014), resulting in a single sinuous (star-shaped) whorl of carpels, with the coils compressed together into zig-zag radial lines. In this case, the three stamens in *Lacandonia* are located at the tips of three upward loops of a sinuous whorl. Regardless of interpretation, obscurity still surrounds the evolutionary derivation of a female flower with carpel fascicles from one without them. We speculate that understanding the basis for this structure could clarify the processes that led to the inside-out phenotype in *Lacandonia*.

The final conundrum in this series is the derivation of the unisexual flowers of Triuridaceae from a “typical” monocot flower, which is trimerous and pentacyclic, with three organs per whorl arranged in five whorls: two tepal whorls, two stamen whorls, and a single central carpel whorl with three fused carpels and septal nectaries (Remizowa, Sokoloff & Rudall, 2010). Most lilioid monocots are relatively conservative in their floral groundplan. In Velloziaceae, the sister family to all other Pandanales (e.g., Mennes et al., 2013), the flowers are at least superficially closer to the typical monocot structure, though some species possess a corona of six petaloid appendages, and others show increased stamen number (Sajo, Mello-Silva & Rudall, 2010).

Conversely, in some Pandanales, loss of the typical monocot flower groundplan is so extensive that precise delimitation of the inflorescence–flower boundary is problematic (Rudall, 2003; Rudall & Bateman, 2006; Rudall, 2008). Like Triuridaceae, flowers are also unisexual in most Cyclanthaceae and Pandanaceae, with highly atypical organ numbers. In *Cyclanthus*, female and male flowers are united in rings, with a loss of flower individuality (Sajo et al., 2014). The female reproductive units of *Sararanga* (Pandanaceae) possess up to 80 carpels united to form a unilocular ovary, and the carpels follow a zigzag arrangement along a folded axis, reminiscent of the asterid eudicot *Tupidanthus* (Araliaceae: Sokoloff et al., 2007). The pseudanthial theory proposed by Rudall (2003) for Triuridaceae was partly dependent on a postulated close relationship with Pandanaceae and Cyclanthaceae. The results of subsequent molecular (Mennes et al., 2013) and morphological (Rudall & Bateman, 2006) cladistic analyses suggesting a closer relationship with Stemonaceae, which have typical bisexual flowers, appeared to contradict this hypothesis. Flowers of Stemonaceae are bisexual but resemble those of Triuridaceae in many respects (Rudall et al., 2005; Rudall & Bateman, 2006). In some Stemonaceae (e.g., *Croomia*), the carpel whorl is reduced to a single carpel, leading Rudall & Bateman (2006) to postulate derivation of the Triuridaceae flower by carpel multiplication. However, a recent whole-plastid genome analysis (albeit with only *Sciaphila* sampled for Triuridaceae) found strong evidence that Triuridaceae is the sister group of a clade comprising Cyclanthaceae and Pandanaceae (Lam, Gomez & Graham, 2015), a topology that would support a loss of developmental homeostasis in these taxa (cf. Rudall & Bateman, 2006).

In conclusion, a homeosis theory for *Lacandonia* origin is not in conflict with any of the various hypotheses for the origin of the Triuridaceae flower, which occurred much earlier in the evolutionary history of this unusual family and coincided with a shift to unisexuality. Indeed, the unisexual yet highly plastic flowers of Triuridaceae could have pre-adapted the origin of the bizarre *Lacandonia* morphology.

## Additional Information and Declarations

### Field Study Permissions

The following information was supplied relating to field study approvals (i.e., approving body and any reference numbers): SisBio collecting permit no. 19769-4 (June 2012–June 2014).

### Data Availability

The following information was supplied regarding data availability: The raw data is presented in the article. Earlier versions of some SEM images (Figs. 10, 11 and 12D) were published previously by one of us (Rudall, 2003; Rudall, 2008), reproduced here with permission from the International Journal of Plant Sciences (University of Chicago Press).

## References

[ref-1] Álvarez-Buylla ER, Ambrose BA, Flores-Sandoval E, Englund M, Garay-Arroyo A, García-Ponce B, De la Torre-Bárcena E, Espinosa-Matías S, Martínez E, Piñeyro-Nelson A, Engström P, Meyerowitz EM (2010). B-Function expression in the flower center underlies the homeotic phenotype of *Lacandoniaschismatica* (Triuridaceae). Plant Cell.

[ref-2] Ambrose BA, Espinosa-Matías S, Vázquez-Santana S, Vergara-Silva F, Martínez E, Márquez-Guzmán J, Álvarez-Buylla ER (2006). Comparative developmental series of the Mexican triurids support a euanthial interpretation for the unusual reproductive axes of *Lacandonia schismatica* (Triuridaceae). American Journal of Botany.

[ref-3] Anderson WR (1980). Cryptic self-fertilization in the Malphiaceae. Science.

[ref-4] Bateman RM, DiMichele WA, Cronk QCB, Bateman RM, Hawkins JA (2002). Generating and filtering major phenotypic novelties: neoGoldschmidtian saltation revisited. Developmental genetics and plant evolution.

[ref-5] Baum DA, Donoghue MJ, Cronk QCB, Bateman RM, Hawkins JA (2002). Transference of function, heterotopy and the evolution of plant development. Developmental genetics and plant evolution.

[ref-6] Cheek M (2003). Kupeaeae, a new tribe of Triuridaceae from Africa. Kew Bulletin.

[ref-7] Endress PK (2014). Multicarpellate gynoecia in angiosperms: occurrence, development, organization and architectural constraints. Botanical Journal of the Linnean Society.

[ref-8] Espinosa-Matías S, Vergara-Silva F, Vázquez-Santana S, Martínez-Zurita E, Márquez-Guzmán J (2012). Complex patterns of morphogenesis, embryology, and reproduction in *Triuris brevistylis*, a species of Triuridaceae (Pandanales) closely related to *Lacandonia schismatica*. Botany.

[ref-9] Franke T, Beenken L, Hahn C (2000). *Triuridopsis intermedia* spec. nov. (Triuridaceae), a new myco-heterotrophic plant from Bolivia. Plant Systematics and Evolution.

[ref-10] Garay-Arroyo P, Piñeyro-Nelson A, García-Ponce B, Sánchez MP, Álvarez-Buylla ER (2012). When ABC becomes ACB. Journal of Experimental Botany.

[ref-11] Hemsley WB (1907). Two new Triuridaceae, with some remarks on the genus *Sciaphila* Blume. Annals of Botany.

[ref-12] Lam VKY, Gomez MS, Graham SW (2015). The highly reduced plastome of mycoheterotrophic *Sciaphila* (Triuridaceae) is colinear with its green relatives and is under strong purifying selection. Genome Biology and Evolution.

[ref-17] Maas H, Maas P, Melo A (2015). http://floradobrasil.jbrj.gov.br/jabot/floradobrasil/FB110684.

[ref-13] Maas PJM, Rübsamen T (1986). Triuridaceae. Flora neotropica, monograph.

[ref-14] Maas-van de Kamer H, Rudall PJ, Cribb PJ, Cutler DF, Humphries CJ (1995). Triuridiflorae: gardner’s delight?. Monocotyledons: systematics and evolution.

[ref-15] Maas-van de Kamer H, Maas PJM (1994). *Triuridopsis*, a new monotypic genus in Triuridaceae. Plant Systematics and Evolution.

[ref-16] Maas-van de Kamer H, Weustenfeld T, Kubitzki K (1998). Triuridaceae. The families and genera of vascular plants. Vol. III. Flowering plants, monocotyledons, Lilianae (except *Orchidaceae*).

[ref-18] Mabberley DJ (2008). Mabberley’s plant book: a portable dictionary of plants, their classification, and uses.

[ref-19] Márquez-Guzmán J, Engleman M, Martínez-Mena A, Martínez E, Ramos C (1989). Anatomía reproductiva de *Lacandonia schismatica* (Lacandoniaceae). Annals of the Missouri Botanical Garden.

[ref-20] Márquez-Guzmán J, Vázquez-Santana S, Engleman EM, Martínez-Mena A, Martínez E (1993). Pollen development and fertilization in *Lacandonia schismatica* (Lacandoniaceae). Annals of the Missouri Botanical Garden.

[ref-21] Martínez SE, Davidse G, Sousa M, Chaters AD (1994). Triuridaceae. Flora mesoamericana, Vol. VI. Alismataceae and cyperaceae.

[ref-22] Martínez SE, Ramos CH (1989). Lacandoniaceae (Triuridales): una nueva familia de México. Annals of the Missouri Botanical Garden.

[ref-23] Melo A, Alves M (2012). The discovery of *Lacandonia* (Triuridaceae) in Brazil. Phytotaxa.

[ref-24] Mennes CB, Smets EF, Moses SN, Merckx VSFT (2013). New insights in the long-debated evolutionary history of Triuridaceae (Pandanales). Molecular Phylogenetics and Evolution.

[ref-25] Merckx VSFT, Freudenstein JV, Kissling J, Christenhusz MJM, Stotler RE, Crandall-Stotler B, Wickett N, Rudall PJ, Maas-van de Kamer H, Maas PJM, Merckx VSFT (2013). Chapter 2: taxonomy and classification. Mycoheterotrophy: the biology of plants living on fungi.

[ref-26] Merckx VSFT, Smets EF, Specht CD, Merckx VSFT (2013). Chapter 3: biogeography and conservation. Mycoheterotrophy: the biology of plants living on fungi.

[ref-27] Miers J (1852). On the family of Triuridaceae. Transactions of the Linnean Society of London.

[ref-28] Philbrick CT (1984). *Pollen tube growth within vegetative tissues* of *Callitriche* (Callitrichaceae). American Journal of Botany.

[ref-29] Remizowa MV, Sokoloff DD, Rudall PJ (2010). Evolutionary history of the monocot flower. Annals of the Missouri Botanical Garden.

[ref-30] Rübsamen-Weustenfeld T (1991). Morphologische, embryologische und systematische Untersuchungen an Triuridaceae. Bibliotheca Botanica.

[ref-31] Rudall PJ (2003). Monocot pseudanthia revisited: floral anatomy and systematics of the mycoheterotrophic family Triuridaceae. International Journal of Plant Sciences.

[ref-32] Rudall PJ (2008). Fascicles and filamentous structures: comparative ontogeny of morphological novelties in Triuridaceae. International Journal of Plant Sciences.

[ref-33] Rudall PJ (2010). All in a spin: centrifugal organ formation and floral patterning. Current Opinion in Plant Biology.

[ref-34] Rudall PJ, Bateman RM (2006). Morphological phylogenetic analysis of Pandanales: testing contrasting hypotheses of floral evolution. Systematic Botany.

[ref-35] Rudall PJ, Bateman RM (2010). Defining the limits of flowers: the challenge of distinguishing between the evolutionary products of simple versus compound strobili. Philosophical Transactions of the Royal Society B.

[ref-36] Rudall PJ, Cunniff J, Wilkin P, Caddick LR (2005). Evolution of dimery, pentamery and the monocarpellary condition in Stemonaceae (Pandanales). Taxon.

[ref-37] Rudall PJ, Remizowa MV, Prenner G, Prychid CJ, Tuckett RE, Sokoloff DD (2009). Non-flowers near the base of extant angiosperms? Spatiotemporal arrangement of organs in reproductive units of Hydatellaceae, and its bearing on the origin of the flower. American Journal of Botany.

[ref-38] Rudall PJ, Sokoloff DD, Remizowa MV, Conran JG, Davis JI, Macfarlane TD, Stevenson DW (2007). Morphology of Hydatellaceae, an anomalous aquatic family recently recognized as an early-divergent angiosperm lineage. American Journal of Botany.

[ref-40] Sajo MG, Lombardi JA, Forzza RC, Rudall PJ (2014). Comparative anatomy of reproductive structures in Cyclanthaceae (Pandanales). International Journal of Plant Sciences.

[ref-39] Sajo MG, Mello-Silva R, Rudall PJ (2010). Homologies of floral structures in Velloziaceae, with particular reference to the corona. International Journal of Plant Sciences.

[ref-41] Sokoloff DD, Oskolski AA, Remizowa MV, Nuraliev MS (2007). Flower structure and development in *Tupidanthus calyptratus* (Araliaceae): an extreme case of polymery among asterids. Plant Systematics and Evolution.

[ref-42] Vergara-Silva F, Espinosa-Matías S, Ambrose BA, Vázquez-Santana A, Martínez-Mena J, Márquez-Guzmán E, Meyerowitz EM, Álvarez-Buylla ER (2003). Inside-out flowers characteristic of *Lacandonia schismatica* (Lacandoniaceae: Triuridales) evolved at least before the divergence from its sister taxon, *Triuris brevistylis*. International Journal of Plant Sciences.

[ref-43] Vogel S (1990). The role of scent glands in pollination: on the structure and function of osmophores.

[ref-44] Wang XF, Tao YB, Lu YT (2002). Pollen tubes enter neighbouring ovules by way of receptacle tissue, resulting in increased fruit-set in *Sagittaria potamogetifolia* Merr. Annals of Botany.

